# A surgical partial excision for the treatment of lingual ectopic thyroid: a case report

**DOI:** 10.11604/pamj.2023.44.46.32565

**Published:** 2023-01-24

**Authors:** Ariefian Erlangga, Muhtarum Yusuf

**Affiliations:** 1 Department of Otorhinolaryngology, Faculty of Medicine, Airlangga University, Dr. Soetomo General Academic Hospital, Surabaya, Indonesia

**Keywords:** Ectopic thyroid, childbirth complications, surgery, health outcome, case report

## Abstract

The lingual ectopic thyroid is a sporadic case. Based on the medical records of Dr. Soetomo General Academic Hospital, Surabaya, they only found one case of ectopic thyroid at least in the last ten years. There is no consensus in the literature about the best therapeutic strategy in managing ectopic thyroid. A 20-year-old female patient with a diagnosis of ectopic lingual thyroid. She has complained of lumps at the base of her tongue since she was ten years old. She performed a partial excision of the tumor with a transoral approach. Partial excision of the lingual ectopic thyroid results in an airway free from obstruction, leaving the rest of the thyroid tissue functioning so that the patient does not require lifelong hormone treatment but has the potential for hypertrophy to recur. The transoral approach provides post-operative results that maintain aesthetic function and reduce morbidity and hospitalization. Partial excision of lingual ectopic thyroid gives good results.

## Introduction

Ectopic thyroid is a condition where thyroid tissue is found not to be where it should be i.e., anterolateral to the second to fourth tracheal rings. Ectopic thyroid has a prevalence rate of 1:100,000 [1,2]. Post-mortem studies have shown that 10% of the population found an ectopic thyroid buried in the thyroglossal duct of all genders [1]. Lingual thyroid is the most common form of ectopic thyroid, accounting for 90% of reported cases [1,3,4]. Management of ectopic thyroid depends on the size of the mass, complaints, and complications [5]. There is no consensus in the literature about the best therapeutic strategy in managing ectopic thyroid because it is a rare case and has limited treatment. The main goal of therapy is to relieve upper airway obstruction [6]. Asymptomatic cases could be treated conservatively without medical or surgical intervention with periodic evaluation. Medical therapy with exogenous hormones treats subclinical hypothyroidism and gland suppression to reduce mass or prevent hypertrophy [7,8]. Patients who do not respond to medical therapy may be considered for surgery. Other indications for surgery, especially at the level of urgency, include dyspnea, dysphonia, dysphagia, suspicion of malignancy, hormonal disturbances, or bleeding [8]. The objective of this study is to report a case of lingual ectopic thyroid that underwent partial surgical excision by a transoral approach. This case report has been reported in line with the SCARE [9].

## Patient and observation

**Patient information:** a female patient of 20 years named Miss. T from Trenggalek, ethnic Javanese-Indonesian, and private employee occupation, came to the Outpatient Unit of Otolaryngology, Dr. Soetomo General Academic Hospital Surabaya with symptoms of a lump at the base of the tongue since ten years ago and it is getting bigger. Another symptom has been more susceptible to coughs and colds since the lump appeared. One month ago, shortness of breath was felt when coughing or doing strenuous activity. There is no sore throat or swallowing pain, hoarse voice, swallowing disorders, or snoring during sleep. No complaints in the ears, nose, or neck. The patient also denied any previous history of severe illness, such as asthma, allergies, diabetes mellitus, and hypertension. The patient does not have a smoking habit. The patient and family admitted that they had not received any intervention before.

**Figure 1:**
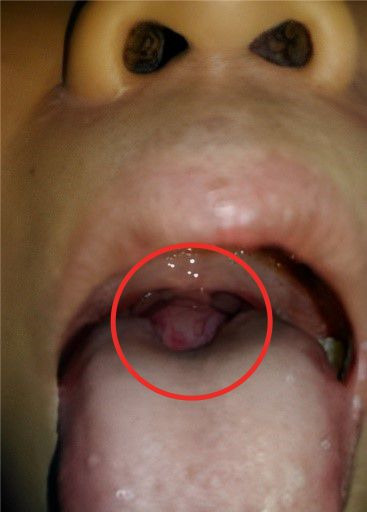
physical examination of the throat revealing a mass in the middle at the base of the tongue, pink (red circle)

**Clinical findings:** on physical examination, it was found to have a pink mass with a smooth surface, a soft solid following the movement of the tongue, not bleeding easily, painless, and vascularized in the middle at the base of the tongue (Figure 1). On palpation of the neck, there was no anterior thyroid tissue

**Timeline of the current episode:** In 2008, patient felt the first symptom in her tongue. On June 2021, she felt worse about her symptom. On July 9^th^ 2018, she came to Dr. Soetomo General Academic Hospital for the first time and diagnosed her symptom. On August 20^th^ 2018, the surgery and thyroid function blood test was done. On August 29^th^ 2018, the histopathological examination for the tumor tissue was out and on September 20^th^ 2018, the last post-operative checkup was done with a good examination test result.

**Figure 2:**
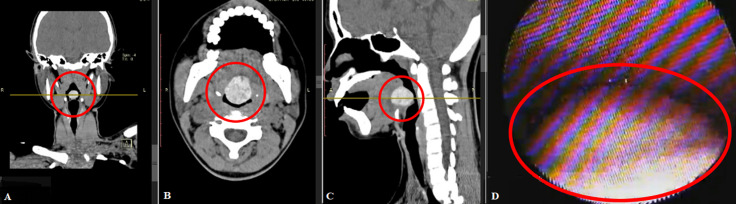
A, B, C) head and neck CT-scan; D) Fiber optic laryngoscope (FOL *)*; there is a soft tissue mass at the base of the tongue (red circle)

**Diagnostic assessment:** the results of a computerized tomography scan (CT scan) of the head and neck showed several outcomes, such as; the impression of a benign, high-density soft tissue mass of 2.6 x 2.2 x 2 cm in the posterior of the tongue, which did not show a contrast enhancement; a mass protruding posteriorly, narrowing the airway to the narrowest diameter of ±0.4 cm, which may represent a lingual thyroid with accumulated iodine in it (Figure 2A, B, C). On examination, the fiber optic laryngoscope (FOL) revealed a unilocular mass, with a smooth, pale color, in contact with the upper edge of the epiglottis, attached to the base of the tongue (Figure 2D). Plain chest radiographs were within normal limits. A blood examination revealed subclinical hypothyroidism (TSH 17,049). The patient was consulted at an endocrinology polyclinic for subclinical hypothyroid evaluation, and there were no contraindications for surgery. The patient is scheduled for partial tumor excision with a transoral approach.

**Diagnosis:** she was diagnosed with ectopic lingual thyroid with subclinical hypothyroidism.

**Figure 3:**
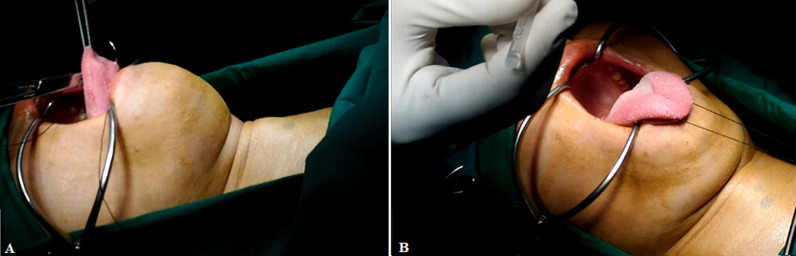
A) suturing the tongue; B) pulling anteroinferior with Kocher

**Figure 4:**
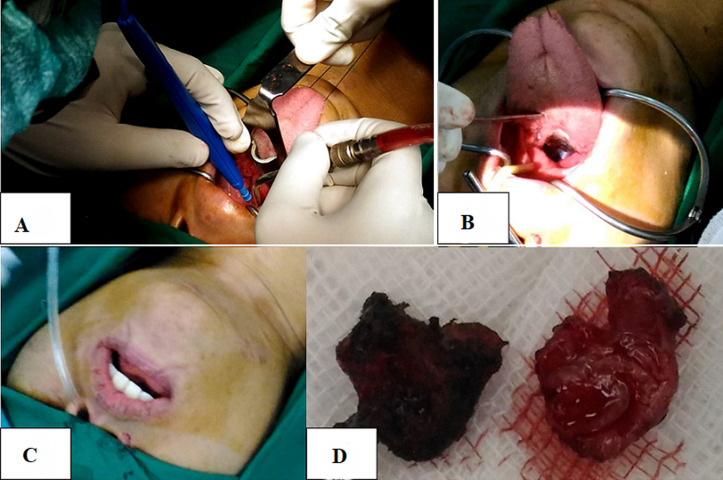
A) excision of the tongue base tumor with cautery; B) the tumor is reduced and appears flush with the tongue's surface; C) nasogastric tube installation; D) the result of excision of a portion of the tumor tissue

**Therapeutic interventions:** partial tumor excision was performed with a transoral approach in the Integrated Surgery Center (GBPT = Gedung Bedah Pusat Terpadu of Dr. Soetomo General Hospital). Preparation for surgery included informed consent (tumor excision, blood transfusion if needed, tracheotomy if needed, post-operative nasogastric tube (NGT) insertion), and the prophylactic antibiotic cefazolin 2 grams intravenously. The operating field was narrowed with a sterile dock, and then a mouth spreader was installed. The tongue is sutured and pulled anteroinferior with a Kocher (Figure 3(A, B)). On initial exploration, a tumor mass was found at the base of the tongue. The mass was cauterized and excised starting at the edge (Figure 4A). The mass was partially excised until it was flush with the tongue's surface (Figure 4B). Bleeding is treated with suction and cautery. A size 14 of NGT was placed in the left nostril for the nutritional intake process for three days of post-operative care (Figure 4C). Based on the operation results, a mass of tumor tissue was found at the base of the tongue, which was sent to the anatomical pathology (AP) section for histopathological examination (Figure 4D). The operation lasted 1 hour without complications, and the amount of bleeding was about 30 ml. Post-operative care patients are advised to rinse with ice water for one week after surgery, complete oral fasting, liquid diet via NGT, metamizole injection 3 x 1 gram intravenously, and antibiotic ceftriaxone injection 2 x 1 gram intravenously. The patient is also scheduled to have a blood test for thyroid function.

**Figure 5:**
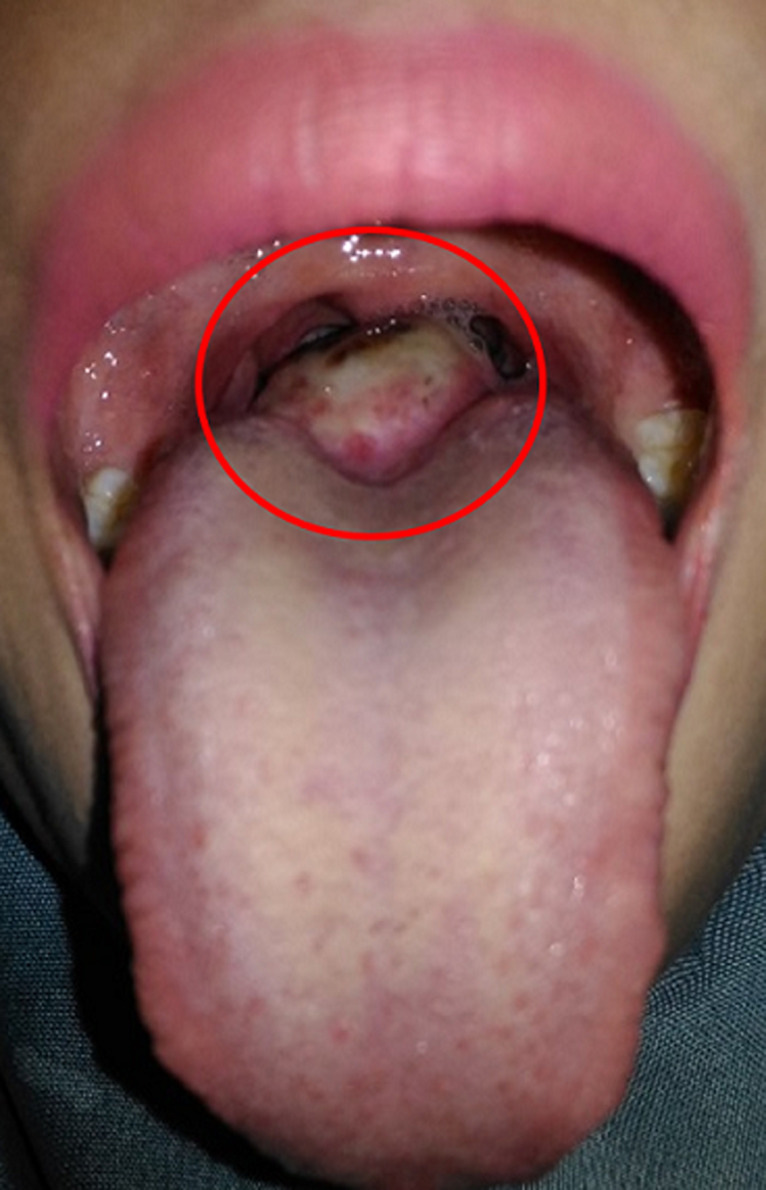
crusting and edema of the surgical wound (red circle)

**Figure 6:**
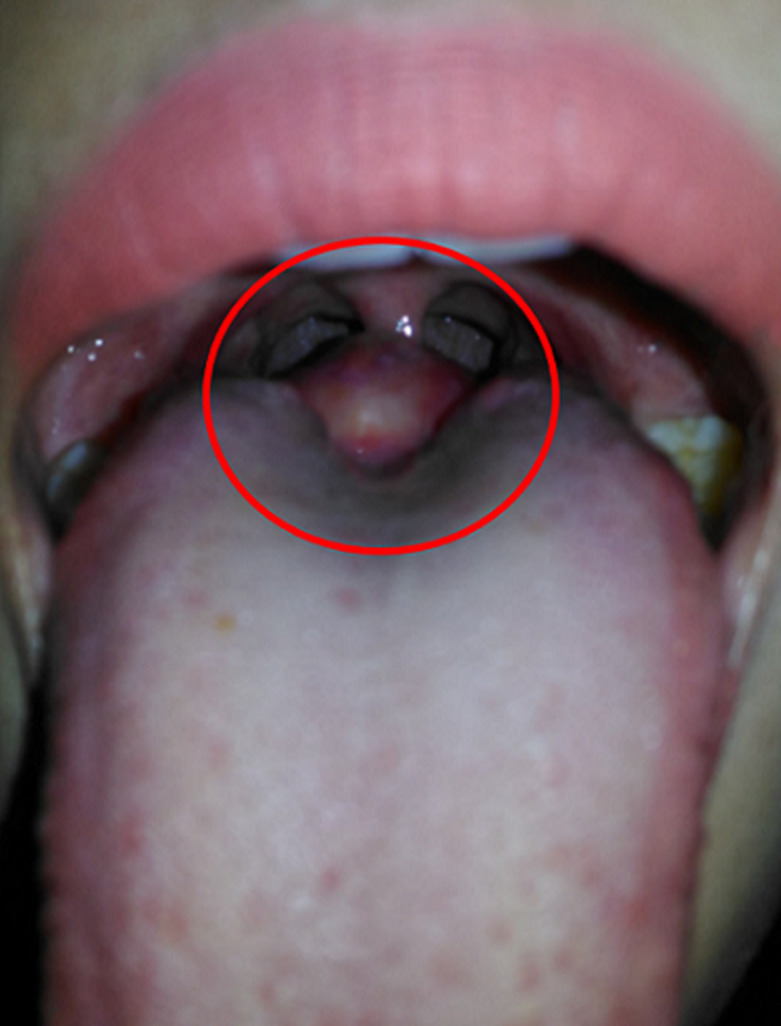
mass at the base of the tongue (red circle)

**Figure 7:**
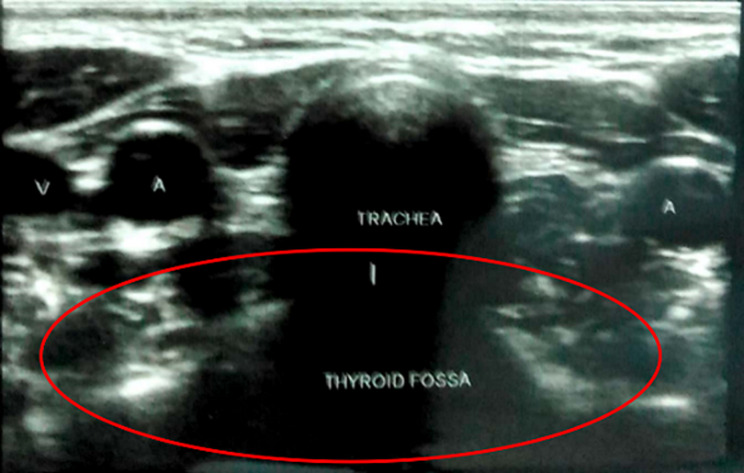
ultrasound of the neck revealed no thyroid formation in the thyroid fossa (red circle)

**Follow-up and outcome of interventions:** one day after surgery, thyroid function blood tests were obtained with subclinical hypothyroidism (TSH 6.46). The department of internal medicine suggested the patient does not require additional thyroid hormone medication, evaluate thyroid function, and control to the endocrinology poly if recharged from the hospital. Three days after surgery, the NGT was removed, and the patient was given a soft diet and advised to avoid eating warm drinks for up to 1 week after surgery. Four days after surgery, the patient's complaints improved, there was no shortness of breath, and the pain in the operating area was reduced. There were crusts and ulcers on the surgical wound (Figure 5). The patient was sent home with mefenamic acid drinking 3 x 500 mg. Two weeks after surgery, the patient came to the outpatient unit and submitted the histopathological examination results to conclude that ectopic thyroid tissue was obtained. There were no complaints of shortness of breath, a feeling of a lump in the throat, coughing, or pain in swallowing rather rough food in the anamnesis. Crusts and udder were found in the surgical wound area on physical examination. The patient was scheduled for an ultrasound examination of the neck. Patients are also being scheduled for thyroid function blood tests from the endocrinologist. One month after surgery, there were no complaints of shortness of breath, no feeling of a lump, no pain, and difficulty swallowing. From the physical examination, it appears that the swelling in the surgical wound has decreased (Figure 6). The neck ultrasound showed no thyroid formation in the right and left thyroid fossa (Figure 7). The results of a blood examination of thyroid function showed subclinical hypothyroidism (TSH 10.901). Patients are advised to have regular check-ups with the endocrinologist for periodic evaluation of thyroid function and control with the otolaryngology poly if there are some symptoms and be informed of the possibility of recurrence associated with increased hormonal needs including during pregnancy and menopause.

**Patient perspective:** “prior to the operation, I had difficulty breathing, and it was so painful to swallow that I did not enjoy the food at all. During the operation process, I was a little scared and tense during the operation, but the doctor did it very well. After the tumor was removed, I felt much better than before”.

**Informed consent:** the patient and her family provided written informed consent to publish her data and any related images.

## Discussion

One case of lingual ectopic thyroid in a 20-year-old woman, ethnic Javanese-Indonesian. Lingual ectopic thyroid is a sporadic disorder [1,2]. Ectopic thyroid tissues are reported in 7-10% of adults, generally females [3]. Clinically, symptoms associated with ectopic thyroid are more common in females. Xavier *et al*. and Prasad *et al*. reported an incidence in females of four to one in males [8,9]. Xavier *et al*. writing in a wide age range from 6 to 74 years, with two peaks at 12 and 50 years old. The mean age of patients reported by Perdoni *et al*. is 40 years, and there is a wide distribution, with peak incidence at puberty, pregnancy, and menopause [1,8]. This physiological state is associated with elevated TSH and the potential for hypertrophy of ectopic thyroid tissue [1].

In this case, partial surgical excision was performed. Treatment of ectopic thyroid depends on the patient's symptoms and thyroid function. Patients with minimal symptoms are advised to be observed (conservative). Hypothyroid patients with lingual thyroid symptoms, which arise due to the mass effect of hypertrophy of the gland, undergo mass reduction when given thyroxine (Rhinitis medicamentosa) [10]. Surgical intervention is required in cases of lingual ectopic thyroid that are refractory to medical therapy. Indications for surgery include dyspnea, dysphagia, dysphonia, or bleeding. Patients are informed about the possibility of permanent hypothyroidism because 70-90% of patients do not have a functional orthotopic gland [1]. Surgery is the most appropriate treatment for patients with ectopic thyroid tissue who show clinical signs of upper airway obstruction or when the lesions show signs of infection or malignant degeneration. Removal of ectopic thyroid tissue in the absence of other thyroid tissue can lead to hypothyroidism, which requires hormonal treatment [11].

Surgery for ectopic thyroid has a variety of advantages and disadvantages. Total thyroidectomy results in thyroid function loss and is replaced by lifelong levothyroxine consumption. In partial excision, there is a risk of re-hypertrophy of the thyroid tissue, especially when metabolic needs increase [12]. Preoperatively, the patient is evaluated for airway clearance, considered for nasotracheal intubation, and possibly requires a tracheostomy. The larger the mass or the presence of a laryngeal mass, the more necessary a tracheotomy or consideration should be given to not releasing nasotracheal intubation for 24 hours postoperatively [13]. Several surgical techniques are mentioned in the literature, including thyroglossal, suprahyoid, and cervical pharyngotomy, but these techniques are less invasive, have a high morbidity rate, interfere with function and aesthetics, and increase recovery time in the hospital. Another alternative is the transoral technique, which provides the advantage of leaving no surgical scars. The transoral excision technique has been widely reported in the literature, but the lack of an operating field with or without a microscope requires long surgical instruments and the risk of difficulty controlling bleeding [5,8].

Post-operative management is needed for an operative. Several things must be monitored during the recovery process, such as the presence of airway obstruction, hypoxia, bleeding, high or low blood pressure, post-operative pain, chills or hypothermia, nausea-vomiting, aspiration, falling out of bed, and the rest of the narcotics. Patient monitoring should include medical and nursing observations, specific statements about the wound or surgical site, all complications, and all changes made to care. Prevention of complications includes early mobilization, adequate nutritional intake, preventing skin damage and pressure sores, and pain management [14,15].

## Conclusion

In conclusion, a 20-year-old female patient diagnosed with lingual ectopic thyroid reportedly underwent partial excision of the tumor with a transoral approach without complications. Surgery in this patient for diagnostic and therapeutic indications. Evaluation to date, four months postoperatively it showed improvement in complaints and no signs of recurrence. Although it is a rare case, knowledge of the treatment is necessary. Therefore, the success of this surgery can be a reference in the treatment of lingual ectopic thyroid cases.
